# Neoadjuvant Chemotherapy Followed by Surgery Versus Surgery Alone for Colorectal Cancer: Meta-analysis of Randomized Controlled Trials

**DOI:** 10.1097/MD.0000000000000231

**Published:** 2014-12-02

**Authors:** Lei Huang, Tuan-Jie Li, Jian-Wen Zhang, Sha Liu, Bin-Sheng Fu, Wei Liu

**Affiliations:** From the Guangdong Provincial Key Laboratory of Liver Disease Research, the Third Affiliated Hospital of Sun Yat-Sen University, Guangzhou 510630, Guangdong Province, China (LH, TJL, WL); Department of Gastrointestinal Surgery, the First Affiliated Hospital of Anhui Medical University, Hefei 230022, Anhui Province, China (LH); Organ Transplantation Center, the Third Affiliated Hospital of Sun Yat-Sen University, Guangzhou 510630, Guangdong Province, China (BSF, JWZ).

## Abstract

Effects of neoadjuvant chemotherapy (NAC) on colorectal cancer (CRC) have been largely studied, while its survival and surgical benefits remain controversial. This study aimed to perform a meta-analysis of randomized controlled trials (RCTs), comparing efficacy and safety of NAC plus surgery with surgery alone (SA) for CRC.

We searched systematically databases of MEDLINE, EMBASE, and the Cochrane Library for RCTs comparing NAC and surgery with SA for treating CRC. References of relevant articles and reviews, conference proceedings, and ongoing trial databases were also screened. Primary outcomes included overall and disease-free survivals, total and perioperative mortalities, recurrence, and metastasis. Meta-analysis was performed where possible comparing parameters using relative risks (RRs). Safely analysis was then performed. Outcomes for stages II and III tumors were also meta-analyzed, respectively. Our study was conducted according to intention-to-treat analysis.

A total of 6 RCTs comparing NAC (n = 1393) with SA (n = 1358) published from 2002 to 2012 were identified. Compared with SA, NAC tended to reduce overall recurrences (21.86% vs 25.15%, RR: 0.70, 95% confidence interval [CI]: 0.32–1.56, *P* = 0.09), and prevent vascular invasion (32.30% vs 43.12%, RR: 0.73, 95% CI: 0.53–1.00, *P* = 0.05); and significantly lowered distant metastasis (15.58% vs 23.80%, RR: 0.66, 95% CI: 0.50–0.86, *P* = 0.002), especially liver metastasis rate (13.00% vs 18.25%, RR: 0.71, 95% CI: 0.51–0.99, *P* = 0.04), and associated with higher incidence of ypT0-2 cases upon resection (13.04% vs 6.42%, RR: 2.36, 95% CI: 1.02–5.44, *P* = 0.04). All other parameters were comparable. NAC-related side-effects were generally mild. NAC mainly benefited patients with stage III disease.

NAC could prevent recurrence and metastasis, associates with better tumor stages upon resection, and potentially impedes vascular invasion among CRC patients. NAC does not contribute to significant survival benefits for CRC, and compares favorably with SA in tumor-free resection rates, nodal status upon resection, and postsurgical complications. This level 1a evidence does not support NAC to obviously outweigh SA in terms of survival and surgical benefits for CRC currently.

## INTRODUCTION

Colorectal cancer (CRC) is one of the most common malignancies, and a leading cause of cancer death worldwide.^1^ It is the third most commonly diagnosed cancer in males and the second in females, with over 1.2 million new cancer cases and over 0.6 million deaths each year, and a 5-year survival rate of around 54%.^2^ Even with adjuvant therapy, which has been extensively studied, the prognosis of advanced tumor is far from satisfactory.^3^ Although neoadjuvant chemoradiotherapy achieves low local recurrence rates, it delays administration of optimal chemotherapy, and does not seem to compromise outcomes.^4^

Neoadjuvant chemotherapy (NAC) has attracted increasing attention as a treatment for CRC.^5^ NAC, which is defined as chemotherapy supplied before operation, has been tested in various studies and proven effective against some malignancies, especially breast carcinoma, while its role for CRC patients remains obscure.^4^ The robust peri-surgical and survival advantages of NAC for CRC are weakly informed with insufficient evidence base. There still exist controversies in many other aspects, like down-staging effect and presence of tumor-free resection margin, which have kept unsolved mainly because differences between NAC and surgery alone (SA) for CRC had been compared mostly in retrospective and observational studies, until the randomized controlled trials (RCTs) included in our study emerged.^6^

To the best of our knowledge, pooled analysis on effectiveness of mere NAC followed by surgery compared with SA for only CRC patients has not been found, and this meta-analysis of RCTs seems to be the first one on this issue. In our study, potential advantages of 2 treatments were quantified using the meta-analytical method. Meta-analysis provides the most convincing evidence when pooling data only from RCTs.^7^ Therefore our study, which is based on Preferred Reporting Items for Systematic Reviews and Meta-Analysis (PRISMA)^8^ guidelines and intention-to-treat analysis, and systematically reviews all relevant high-quality RCTs, creates the highest level of evidence.

## MATERIALS AND METHODS

### Ethical Consideration

Since this is a meta-analysis article, and each included publication has appropriate and complete ethical statement, ethical approval was not necessary for this paper.

### Literature Retrieval

**FIGURE 1:**
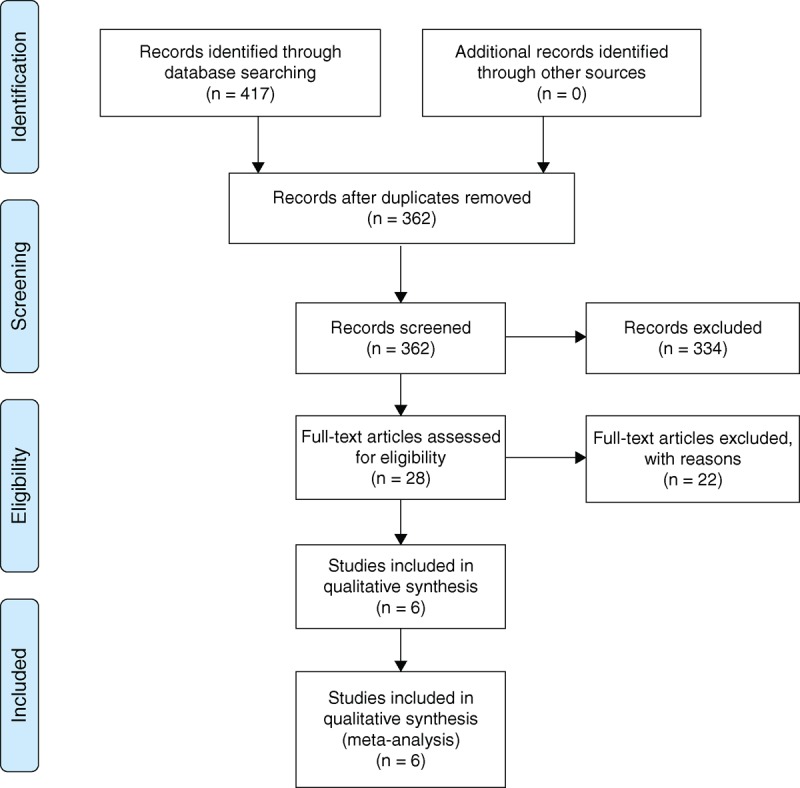
PRISMA literature selection flow diagram. NAC = neoadjuvant chemotherapy, SA = surgery alone, RCT = randomized controlled trial, PRISMA = Preferred Reporting Items for Systematic Reviews and Meta-Analysis.

A systematic literature retrieval with search terms “neoadjuvant/preoperative chemotherapy,” “surgery,” and “colon/rectal/colorectal carcinoma/cancer,” and their combinations as keywords was conducted using MEDLINE, EMBASE, and the Cochrane Library databases, and Google Scholar (Figure 1). Special database functions like “related publications” and “explosion” were applied to maximize our search, and references from relevant articles, cross-references, and reviews were also screened. We also searched conference proceedings and ongoing trial databases. Language restrictions were not applied. The latest search was performed on May 29th, 2014.

### Inclusion Criteria

Titles and abstracts of all identified articles were screened and we selected studies according to the following criteria: population—patients with CRC without age, gender, and racial limitations; intervention and comparative intervention—clearly documented NAC versus SA for CRC, regardless of detailed NAC regimen, timing, modality of administration, duration of chemotherapy, interval between randomization and surgery, surgical method applied, and classification, grade, and position of the lesion; outcomes—at least one of the outcome measures reported below; study design—published and unpublished RCTs. If 2 studies from the same institution were identified, the most recent or the most informative was selected, unless they were reports from different periods or if the data of overlapping patients could be subtracted.

### Exclusion Criteria

Studies were excluded from our analysis if they did not meet the abovementioned inclusion criteria, or the study population included diseases other than CRC (eg, ulcerative colitis, polyps, and polyposis) or neoadjuvant therapy sets other than mere NAC (eg, neoadjuvant radiotherapy and neoadjuvant chemoradiotherapy) unless the data were separately presented, or it was impossible to extract or to calculate appropriate data from the published results.

### Types of Interventions

Any method of chemotherapy performed initially pre-surgery, with or without further postsurgical therapy (if there existed, then the postoperational management, including regimen, administration route, and dose, had to be matched between 2 groups) was included and referred as NAC, regardless of the specific regimen, administration and dosage. As SA we considered all procedures as “primary surgery” or “surgery alone” and merely performed through open operation. Studies with postsurgical therapy comparable between 2 groups applied to guarantee the treatment efficacy were not excluded. Studies that included other types of malignancies or operation (eg, laparoscopic surgery), or those that contained multivisceral resections were excluded unless the data were separatively presented.

### Outcomes of Interest and Definitions

Primary outcomes included 3- and 5-year overall survival (OS) and disease-free survival (DFS) rates, total and perioperative mortalities, recurrence, and metastasis at the end of follow-up. Secondary outcomes were tumor conditions upon resection, including tumor (ypT0-2) and nodal classifications (ypN0), and vascular invasion, curative resection rate, and post-surgical complications. Safety and toxicity analysis focused on adverse effects of NAC was also performed. As described in the included trials, OS was based on survivors during the time from operation to death from any cause, and DFS was according to survivorship during the time until the first relapse of disease. Tumor and nodal classifications upon resection were recorded based on the Union for International Cancer Control (UICC) tumor node metastatic (TNM) classification of malignant tumors.^9^ The clinicopathological responses of the resected specimens were described by Quirke classification.^10^ Toxicity grading was evaluated according to the Common Terminology Criteria for Adverse Events of the National Cancer Institute.^11^

### Data Extraction

Titles and abstracts of all retrieved records, and subsequently full-text articles were examined independently by 2 authors (LH and TJL). The following data were extracted separately by the same 2 authors for all enrolled studies: references of study, characteristics of study population, study design, and inclusion and exclusion criteria. For dichotomous outcomes, we recorded the number of events. Population characteristics included number of participating subjects, regimen of NAC applied, age, and gender. In case of discrepancies, a third author (WL) was consulted and agreement was reached by consensus.

### Risk of Bias Assessment

Risk of bias was evaluated for all articles by individual components using both the Jadad scoring system^12^ and the Cochrane Collaboration's tool for assessing risk of bias. High-quality trials scored more than 2 out of a maximum possible score of 5, while low-quality trials scored 2 or less.

### Statistical Analysis

This study was carried out in the light of the recommendations of the PRISMA^8^ statement. Statistical analyses were performed according to the recommendations of the Cochrane Collaboration Guidelines.^13^ Outcomes reported by 2 or more studies were pooled in meta-analyses. Our study was based on intention-to-treat analysis.

Dichotomous outcomes were presented as relative risks (RRs). Data were pooled using the Mantel–Haenszel method. Trials with zero events in both arms were excluded from meta-analyses. For all analyses, the 95% confidence interval (CI) was quantified. Heterogeneity was assessed using Higgins χ^2^ test,^14^ and inconsistency in study effects was quantified by *I*^2^ values.^15^ The fixed-effects model was applied if no heterogeneity was presented (χ^2^*P* > 0.100 and *I*^2^ < 50%). If excessive heterogeneity existed, data were first rechecked and the DerSimonian random-effects model was applied when heterogeneity persisted.^16^ Funnel plots were drawn to help identify the presence of publication or other types of biases.^17^ Subgroup analysis was planned for studies with single regimen and combination regimens after the overall analysis.^18^ Review Manager software (RevMan© v. 5.0) provided by the Cochrane Collaboration was used for data management and statistical analyses.

## RESULTS

### Selected RCT Characteristics

A total of 6 original RCTs^19–24^ comparing NAC with SA for treating CRC and meeting the eligibility criteria were identified. They were published between 2002 and 2012, with 36 to 73 months for follow-up period. A total of 2751 patients were enrolled in our analysis with 1393 (50.6%) receiving NAC and 1358 (49.2%) receiving SA. Patients’ characteristics are detailed in Table 1. Matching of demographic factors was almost complete and all studies were adequately matched in the factors reviewed (Table 1). All patients had proofs of CRC according to pathology and/or signs and/or symptoms and pre-surgical laboratory and imaging studies (Table 2). Before operation, NAC and SA groups did not diverse significantly in terms of age (58.50 vs 58.74, *Z* = 0.63, *P* = 0.53), gender (male percentage, 59.12% vs 59.14%, *Z* = 0.10, *P* = 0.92), differentiation grade (well [37.64% vs 40.47%, *Z* = 1.22, *P* = 0.22], moderately [47.39% vs 47.01%, *Z* = 0.17, *P* = 0.87], poorly [14.96% vs 12.43%, *Z* = 1.58, *P* = 0.11]), location of primary tumor (ascending colon [24.12% vs 20.56%, *Z* = 0.40, *P* = 0.69], transverse colon [6.64% vs 6.00%, *Z* = 0.38, *P* = 0.70], descending colon [7.81% vs 7.92%, *Z* = 0.01, *P* = 0.99], sigmoid colon [21.48% vs 23.34%, *Z* = 1.04, *P* = 0.30], rectum [40.04% vs 42.18%, *Z* = 0.39, *P* = 0.70]), or follow-up month (48.04 vs 44.92, *Z* = 0.68, *P* = 0.49).

**TABLE 1:**
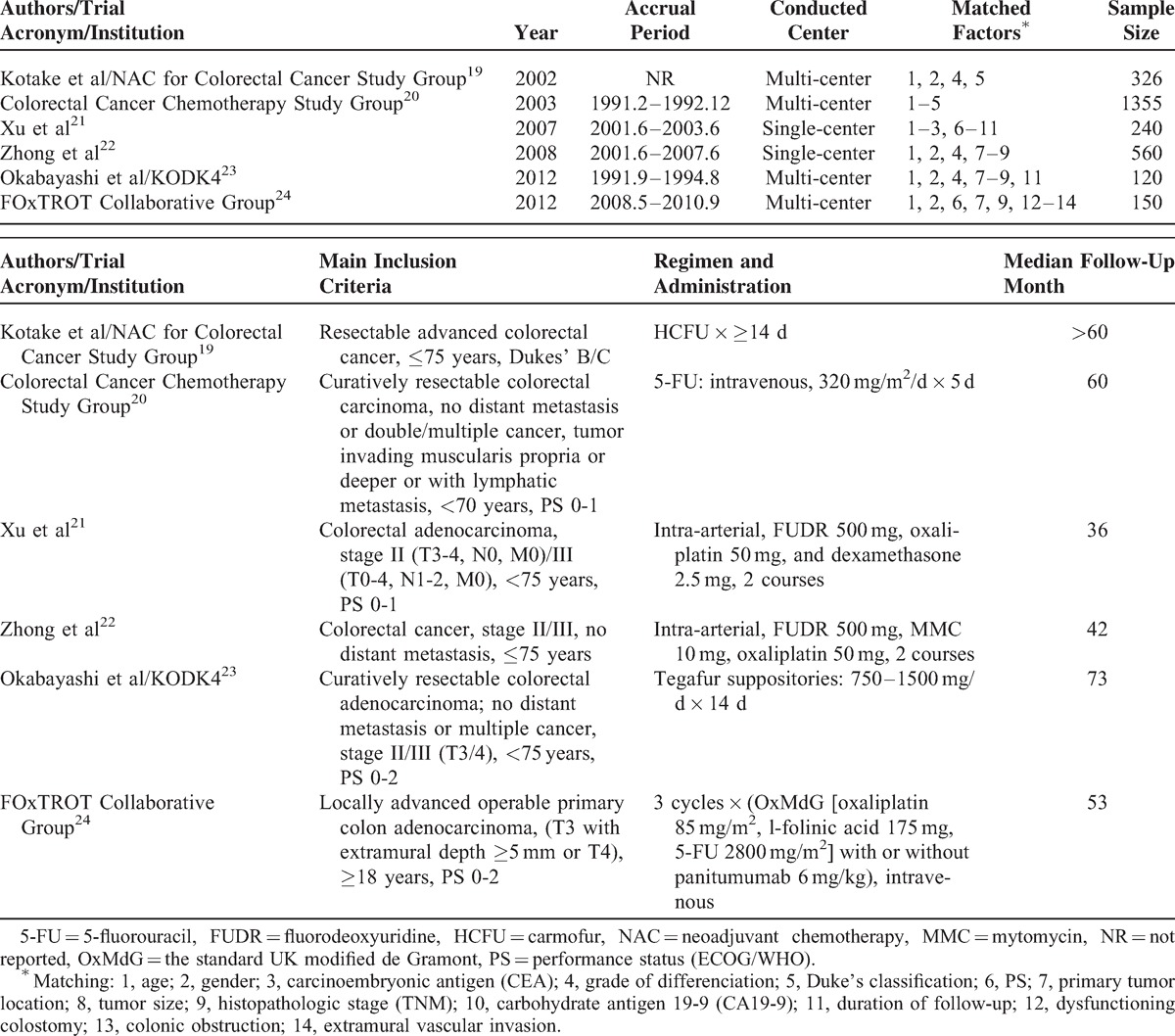
Details of Included Trials in This Meta-Analysis

**TABLE 2:**
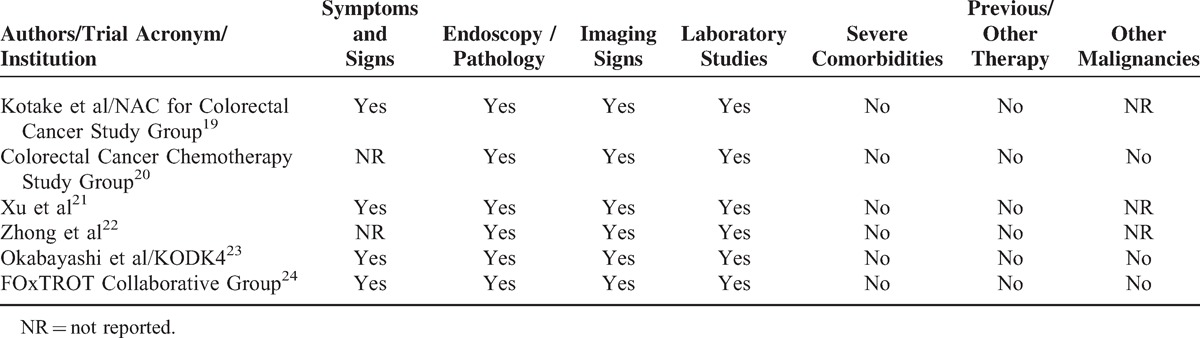
Criteria for Colorectal Cancer Inclusion Eligibility and Assessment

### Methodological Quality Evaluation

The included trials had good methodological qualities with a mean Jadad score of 3 (range, 2–4). They mostly suffered from methodological flaws frequently existing in clinical RCTs generally, majorly, difficulties in concealment of the patients’ allocation, and the inherent complication of blinding between 2 managements. Three trials did not report allocation concealment. All trials had adequate sequence generation, reports on postoperative protocol and loss to follow-up, and sample size calculation (Table 3).

**TABLE 3:**
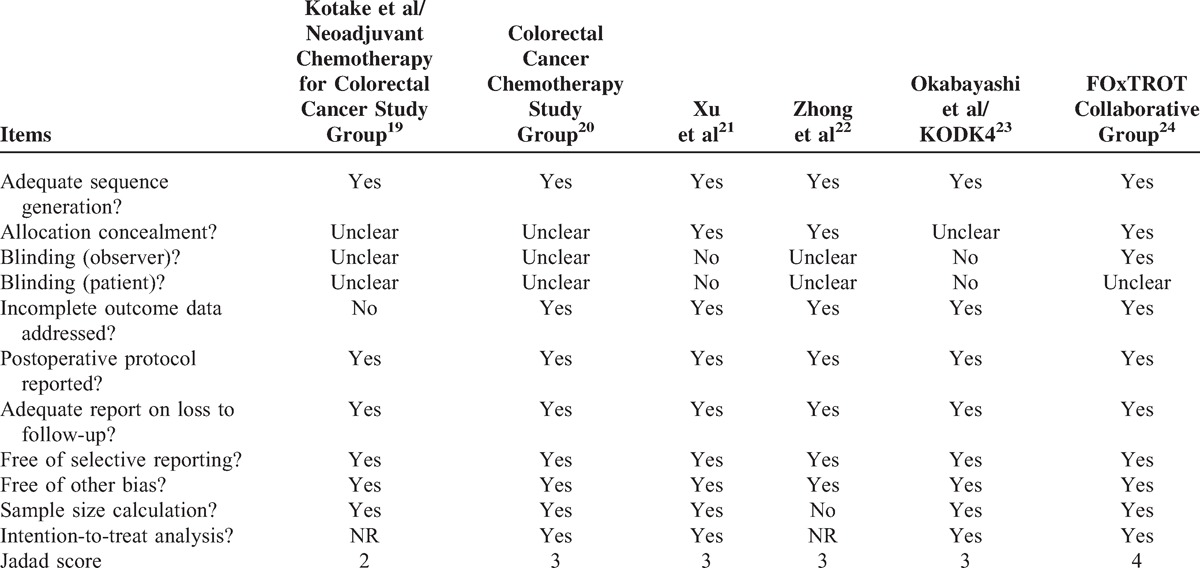
Quality Assessment and Risk of Bias Summary

### Primary Outcomes

Detailed analyses and data by categories are shown in Tables 4 and 5.

**TABLE 4:**
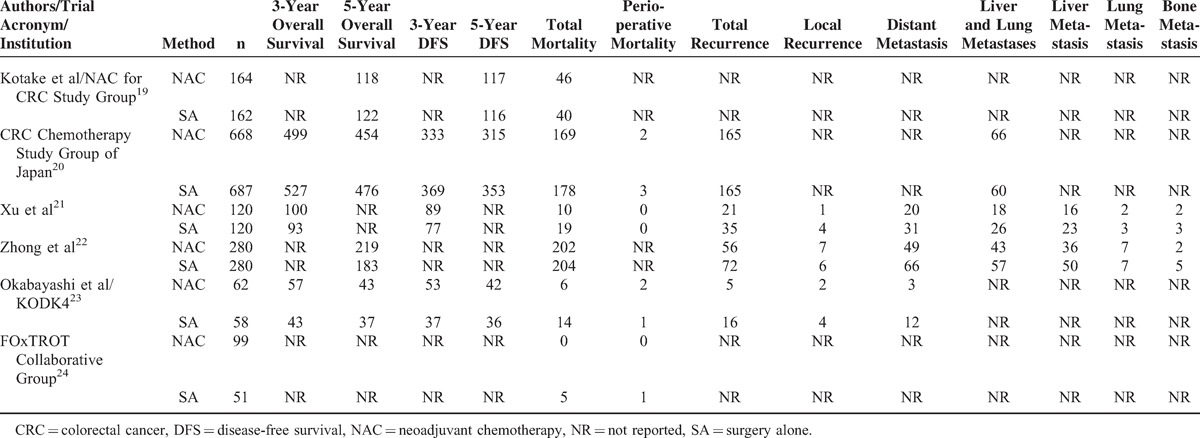
Primary Outcomes

**TABLE 5:**
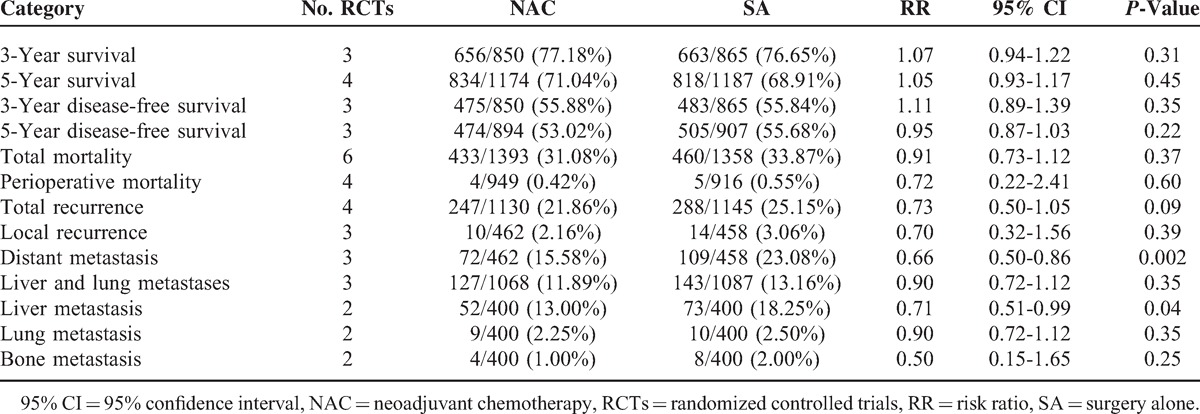
Analysis of Primary Outcomes by Categories

### OS

**FIGURE 2:**
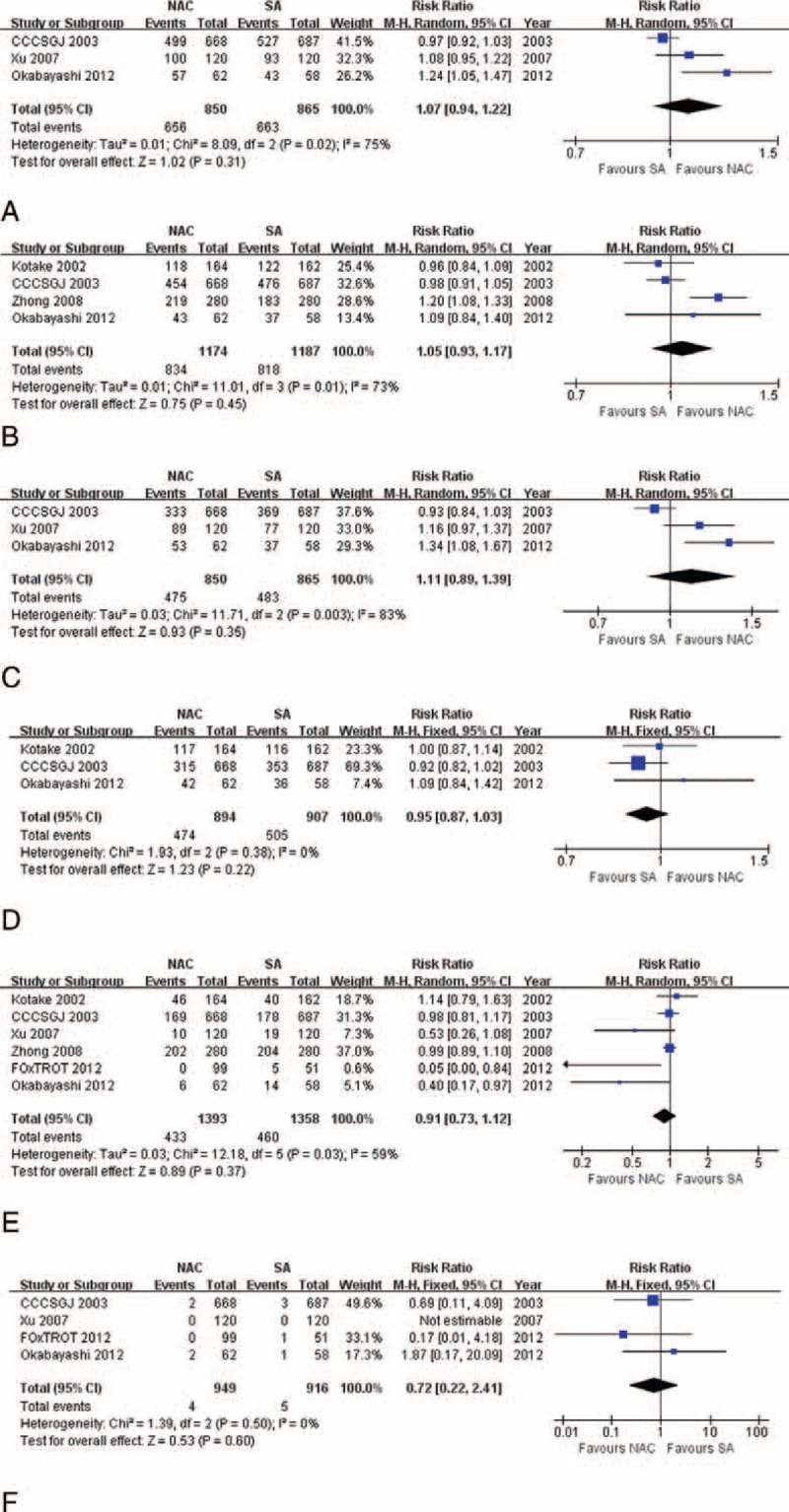
Forest plots for (A) 3-year survival, (B) 5-year survival, (C) 3-year disease-free survival, (D) 5-year disease-free survival, (E) overall mortality, and (F) perioperative mortality by NAC and SA procedures, all showing no significant difference. The relative weight of each study is proportional to the size of the corresponding box in the Forest plot. NAC = neoadjuvant chemotherapy; SA = surgery alone.

Results for 3 and 5 years were available for 3 and 4 RCTs, respectively. Both had significant heterogeneities (χ^2^ = 8.09, *P* = 0.02, *I*^2^ = 75%; χ^2^ = 11.01, *P* = 0.01, *I*^2^ = 73%) between NAC and SA arms, so random-effects model was selected. No significant difference was presented for both parameters between 2 groups for treating CRC (77.18% vs 76.65%, RR: 1.07, 95% CI: 0.94–1.22, *P* = 0.31, Figure 2A; 71.04% vs 68.91%, RR: 1.05, 95% CI: 0.93–1.17, *P* = 0.45, Figure 2B). Kotake et al^19^ reported that survival rates of 2 groups were not affected by either cancer site or nodal status. The Colorectal Cancer Chemotherapy Study Group of Japan (CCCSGJ)^20^ found that even after adjusting for non-curable resection rate, there was no significant difference in the 5-year survival rate, and further revealed no significant differences between 2 procedures for colon and rectal malignancies, respectively.

### DFS

**FIGURE 3:**
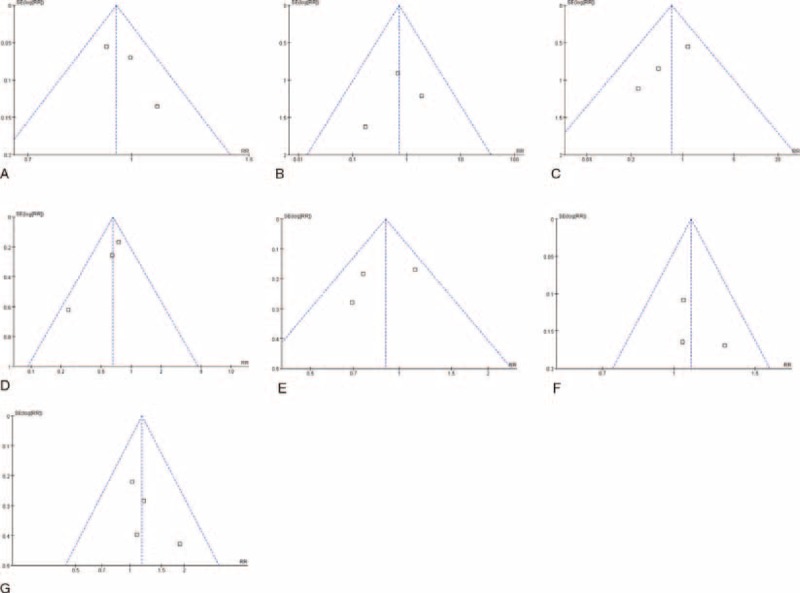
Funnel plots for (A) 5-year disease-free survival, (B) perioperative mortality, (C) local recurrence, (D) distant metastasis, (E) liver and lung metastases, (F) TNM stage upon resection, and (G) postoperative complications, showing that all parameters are free from significant bias. RR = relative risk, SE = standard error.

Result at 3 years based on 3 trials indicated no significant difference between 2 groups (55.88% vs 55.84%, RR: 1.11, 95% CI: 0.89–1.39, *P* = 0.35, Figure 2C) with random-effects model used due to significant heterogeneity (χ^2^ = 11.71, *P* = 0.003, *I*^2^ = 83%). Fixed-effects model applied because of insignificant heterogeneity also suggested no significant difference in 5-year DFS rate (53.02% vs 55.68%, RR: 0.95, 95% CI: 0.87–1.03, *P* = 0.22, Figure 2D), with funnel plot demonstrating no significant bias (Figure 3A). Xu et al^21^ further reported that the preoperative hepatic and regional arterial chemotherapy (PHRAC) arm demonstrated a significantly better liver metastasis-free survival rate compared with the control arm at 3 years (85.5% vs 79.5%, *P* = 0.04), and that the median liver metastasis detection time was significantly longer in the NAC group (19 ± 3 vs 10 ± 2 months, *P* = 0.025).

### Mortality

There being significant heterogeneity (χ^2^ = 12.18, *P* = 0.03, *I*^2^ = 59%), random-effects model selected revealed that there was not significant difference in mortality rates between patients receiving NAC and those undergoing SA at the end of follow-up (6 RCTs, 31.08% vs 33.87%, RR: 0.91, 95% CI: 0.73–1.12, *P* = 0.37, Figure 2E). Perioperative mortality was further analyzed, also revealing comparable results between 2 procedures, with fixed-effects model applied due to insignificant heterogeneity (4 RCTs, 0.42% vs 0.55%, RR: 0.72, 95% CI: 0.22–2.41, *P* = 0.60, Figure 2F), and with funnel plot supporting insignificant bias (Figure 3B).

### Recurrence and Metastasis

**FIGURE 4:**
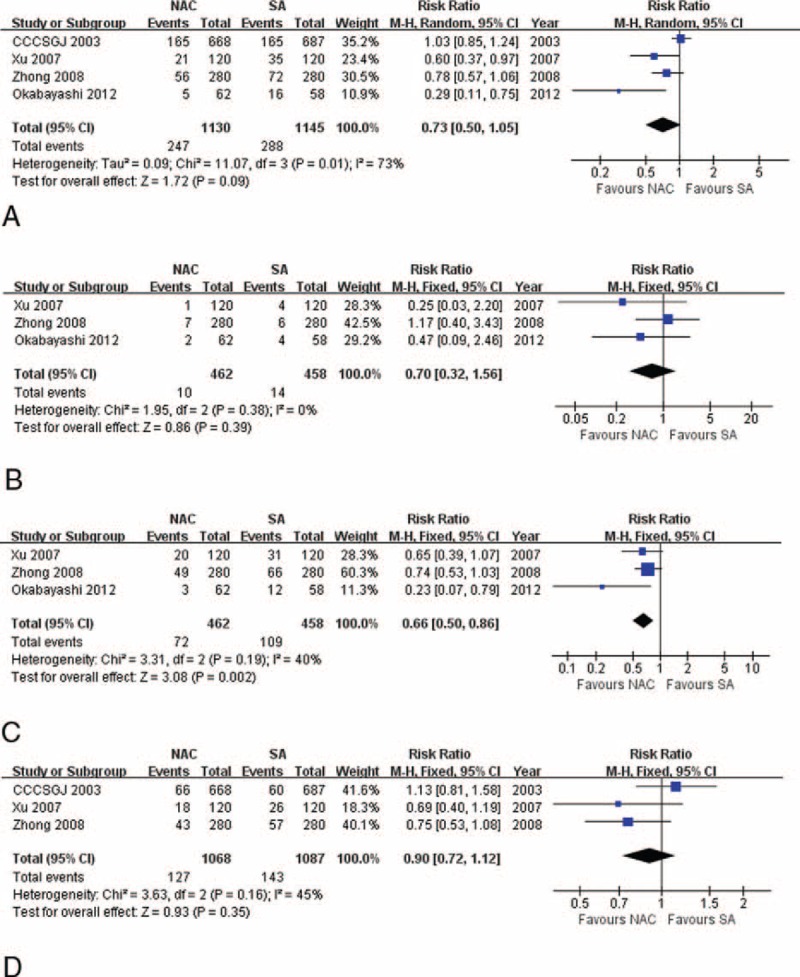
Forrest plots for (A) overall recurrence, showing that NAC tends to lower recurrence rate compared with SA; (B) local recurrence, showing comparable results between NAC and SA processes; (C) distant metastasis, showing NAC significantly reduces distant metastasis rate compared with SA; and (D) liver and lung metastases, showing comparable results between NAC and SA procedures. The relative weight of each study is proportional to the size of the corresponding box in the Forest plot. NAC = neoadjuvant chemotherapy, SA = surgery alone.

Significant heterogeneity was observed (χ^2^ = 11.07, *P* = 0.01, *I*^2^ = 73%), and randomized-effects model selected revealed that NAC tended to contribute to lower recurrence rate than SA (4 RCTs, 21.86% vs 25.15%, RR: 0.70, 95% CI: 0.32–1.56, *P* = 0.09, Figure 4A). Through further analysis, we found that no significant difference existed in local recurrence between 2 groups (3 RCTs, 2.16% vs 3.06%, RR: 0.70, 95% CI: 0.32–1.56, *P* = 0.39, Figure 4B). However, NAC significantly reduced distant metastases compared with SA (3 RCTs, 15.58% vs 23.80%, RR: 0.66, 95% CI: 0.50–0.86, *P* = 0.002, Figure 4C). We further uncovered that the overall preventive effect of liver and lung metastases did not vary significantly between 2 processes (3 RCTs, 11.89% vs 13.16%, RR: 0.90, 95% CI: 0.72–1.12, *P* = 0.35, Figure 4D), and that the lung metastasis rates were comparable between 2 arms (2 RCTs, 2.25% vs 2.50%, RR: 0.90, 95% CI: 0.37–2.19, *P* = 0.82), as well as bone metastasis (2 RCTs, 1.00% vs 2.00%, RR: 0.50, 95% CI: 0.15–1.65, *P* = 0.25). But NAC significantly contributed a prophylactic effect to liver metastasis compared to SA (2 RCTs, 13.00% vs 18.25%, RR: 0.71, 95% CI: 0.51–0.99, *P* = 0.04). All the above parameters were based on fixed-effects model because of insignificant heterogeneity, with funnel plots showing no significant bias (Figure 3C–E). Xu et al^21^ also reported that recurrences in the peritoneum were significantly reduced in the NAC arm. CCCSGJ^20^ further found that initial recurrence most commonly involved the liver in colon cancer, followed by lungs; local recurrence was more common in rectal cancer, with a similar incidence of metastases to the liver and lungs.

### Secondary Outcomes

Detailed analyses and data by categories are shown in Tables 6 and 7.

**TABLE 6:**
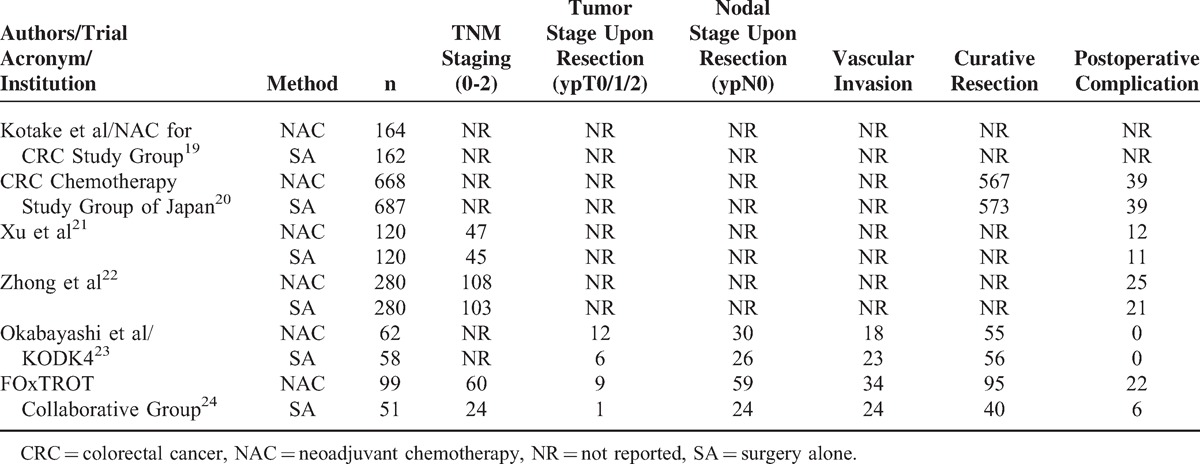
Secondary Outcomes

**TABLE 7:**
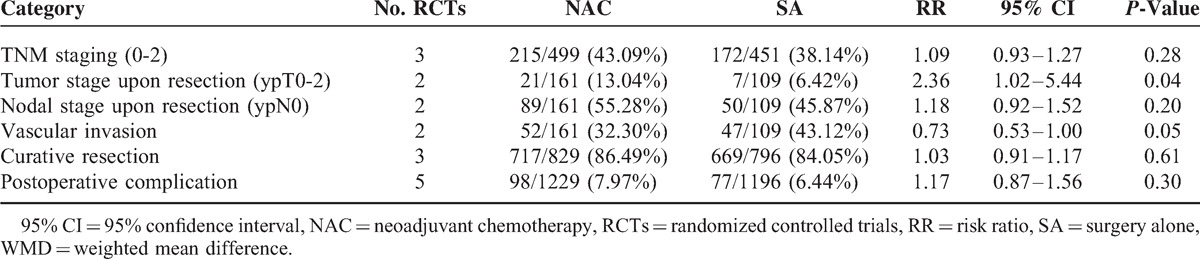
Analysis of Secondary Outcomes by Categories

### Tumor Conditions Upon Resection

**FIGURE 5:**
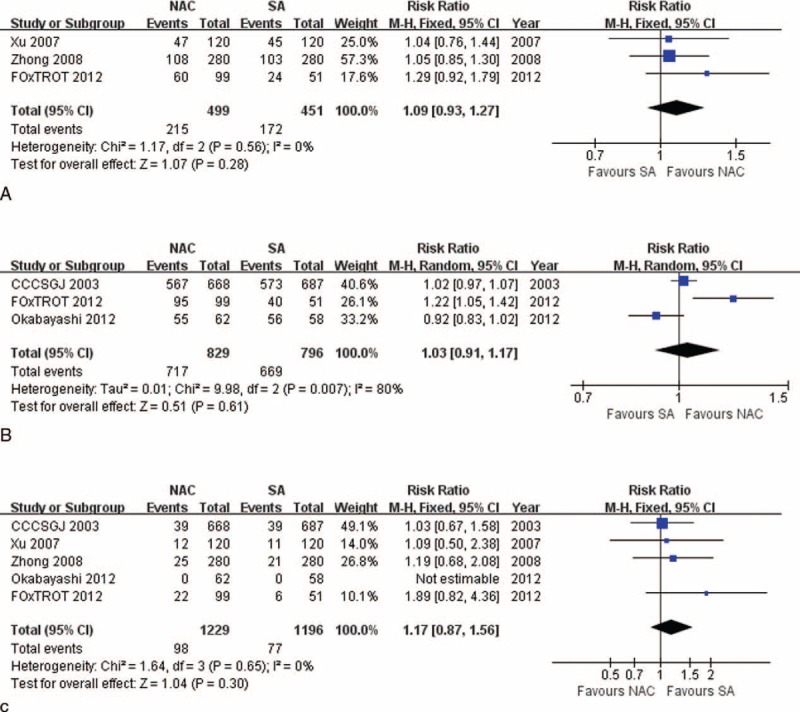
Forest plots for (A) TNM stage upon resection, (B) presence of tumor-free resection margin, and (C) postoperative complications, all showing comparable results between NAC and SA processes. The relative weight of each study is proportional to the size of the corresponding box in the Forest plot. NAC = neoadjuvant chemotherapy, SA = surgery alone.

Since there were not significant heterogeneities, fixed-effects model was used. The combined data revealed similar results for tumor TNM classification (3 RCTs, 43.09% vs 38.14%, RR: 1.09, 95% CI: 0.93–1.27, *P* = 0.28, Figure 5A), and nodal classification (ypN0) (2 RCTs, 55.28% vs 45.87%, RR: 1.18, 95% CI: 0.92–1.52, *P* = 0.20) upon resection. However, pooled result showed that there were significantly more ypT0-2 statuses (2 RCTs, 13.04% vs 6.42%, RR: 2.36, 95% CI: 1.02–5.44, *P* = 0.04) observed upon resection among patients treated with NAC than SA, and that there tended to be fewer vascular invasions in NAC group compared with SA group (2 RCTs, 32.30% vs 43.12%, RR: 0.73, 95% CI: 0.53–1.00, *P* = 0.05). Funnel plots revealed low bias for the above parameters (Figure 3F). FOxTROT Collaborative Group^24^ further reported significant differences favoring NAC in apical node (1.02% vs 20.00%, *P* < 0.001), and retroperitoneal margin involvements (5.32% vs 18.18%, *P* = 0.02). According to that study, the depth of spread beyond the muscularis propria (12.8 ± 8.4 to 9.0 ± 7.9 mm, *P* = 0.002) and the maximum tumor thickness (24.9 ± 12.2 to 19.0 ± 12.8 mm, *P* = 0.002) were reduced compared with baseline in NAC group, which was more obvious than the SA group. NAC significantly reduced moderate or greater regression (31% vs 2%, *P* < 0.001).

### Presence of Tumor-Free Resection Margin

There being significant heterogeneity (χ^2^ = 9.98, *P* = 0.007, *I*^2^ = 80%), analysis with a random-effects model supported that NAC did not hopefully contribute to a significantly higher incidence of curative resection compared with SA (3 RCTs, 86.49% vs 84.05%, RR: 1.03, 95% CI: 0.91–1.17, *P* = 0.61, Figure 5B). However, CCCSGJ^20^ reported significantly greater number of cases of non-curative resection among colon cancer patients in the SA group.

### Complications

Funnel plot indicating no bias (Figure 3G) and heterogeneity not existing, fixed-effects model revealed that postsurgical morbidities between 2 groups were similar (5 RCTs, 7.97% vs 6.44%, RR: 1.17, 95% CI: 0.87–1.56, *P* = 0.30, Figure 5C). Moreover, CCCSGJ^20^ reported no statistically significant difference in postoperative complications requiring treatment (grade G2 or above) was noted between 2 groups. FOxTROT Collaborative Group^24^ revealed that no significant differences were found in complications prolonging hospital stay, procedures resulting in a stoma or further abdominal surgery needed.

### Outcomes for Stage II and III Tumors, Respectively

Two RCTs^21,22^ reporting relevant parameters were separately analyzed. Detailed analyses and data by categories are shown in Table 8.

**TABLE 8:**
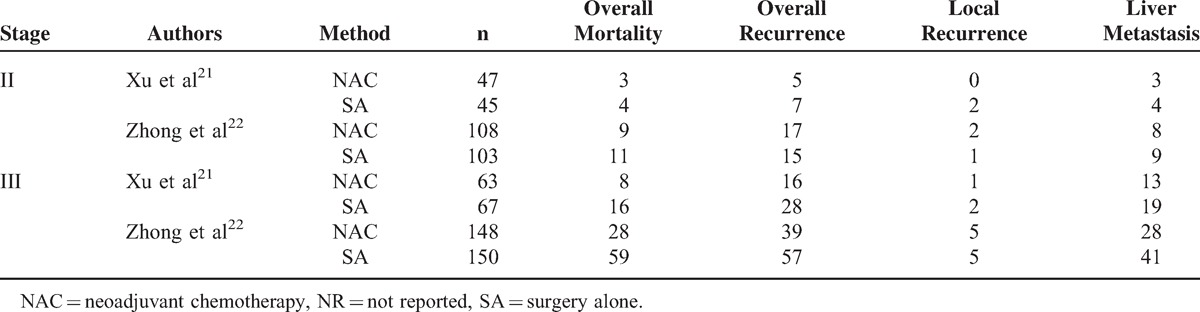
Outcomes for Stage II and III Tumors, Respectively

### Stage II

Total mortality (7.74% vs 10.14%, RR: 0.76, 95% CI: 0.37–1.58, *P* = 0.47), overall recurrence (14.19% vs 14.86%, RR: 0.95, 95% CI: 0.55–1.65, *P* = 0.87), local recurrence (1.29% vs 2.03%, RR: 0.68, 95% CI: 0.14–3.42, *P* = 0.64), and liver metastasis (7.10% vs 8.78%, RR: 0.81, 95% CI: 0.37–1.75, *P* = 0.59) rates were all comparable between 2 groups. Fixed-effects model was used for all the above items due to insignificant heterogeneities. According to Xu et al^21^, there were no significant differences in overall DFS or liver metastasis-free survival rate at 3 years; furthermore, there were also no significant differences in the median liver metastasis detection time noted between the 2 treatment arms.

### Stage III

NAC significantly reduced overall mortality (17.06% vs 34.56%, RR: 0.49, 95% CI: 0.35–0.70, *P* < 0.001) and recurrence rates (26.07% vs 39.17%, RR: 0.67, 95% CI: 0.50–0.88, *P* = 0.005), and tended to prevent liver metastasis (19.43% vs 27.65%, RR: 0.70, 95% CI: 0.50–1.00, *P* = 0.05) compared with SA. However, NAC did not contribute to a significant reduction in local recurrence with compared to SA (2.84% vs 3.23%, RR: 0.88, 95% CI: 0.30–2.57, *P* = 0.81). Fixed-effects model was applied for all the above parameters due to insignificant heterogeneities. According to Xu et al^21^, the RR for OS was 0.51 (95% CI: 0.32–0.67, *P* = 0.009) in the NAC arm; NAC significantly improved DFS rate (74.6% vs 58.1%, *P* = 0.01), and the RR for DFS was 0.61 (95% CI: 0.51–0.79, *P* < 0.001) in the NAC arm; the liver metastasis-free survival rate at 3 years was significantly higher in the NAC group, and the RR for liver metastasis-free survival rate was 0.73 (95% CI: 0.52–0.86, *P* = 0.02) in the NAC arm; furthermore, the median liver metastasis detection time for patients was significantly longer in the PHRAC group compared to SA group (16 ± 3 vs 8 ± 1 months, *P* = 0.01).

### Others

Okabayashi et al^23^ suggested that the preoperative administration may have some cytotoxic potential for preventing tumor recurrence. Xu et al's^21^ histopathologic evaluation after PHRAC showed obvious necrosis in the middle of the tumor lesions as well as in involved lymph nodes. Zhong et al^22^ reported that NAC could restrain proliferation, promote apoptosis and necrosis in CRC. Pooled analyses were not available for these parameters.

### Objective Response to NAC

Okabayashi et al^23^ revealed that 33% patients had responded to the preoperative administration of tegafur suppositories. FOxTROP Collaborative Group^24^ reported preoperative therapy resulted in 2.13% complete pathological responses.

### Safety Analysis

Safety analysis included NAC-related adverse effects. CCCSGJ^20^ reported that adverse reactions were generally mild, and that the most common NAC-related adverse effect was leucopenia. Xu et al^21^ reported that grade 3 hepatic toxicity was observed in only 2% of patients in the PHRAC group and could be lightened before surgery. Okabayashi et al^23^ reported that none of the patients in NAC group experienced any severe adverse effects (≥grade 3), indicating that this treatment was feasible and tolerable. FOxTROP Collaborative Group^24^ reported 34% of patients receiving NAC had grade 3 or worse toxicity, with grade 3–4 gastrointestinal toxicity in 7% of patients in the NAC group.

### Sensitivity Tests

**FIGURE 6:**
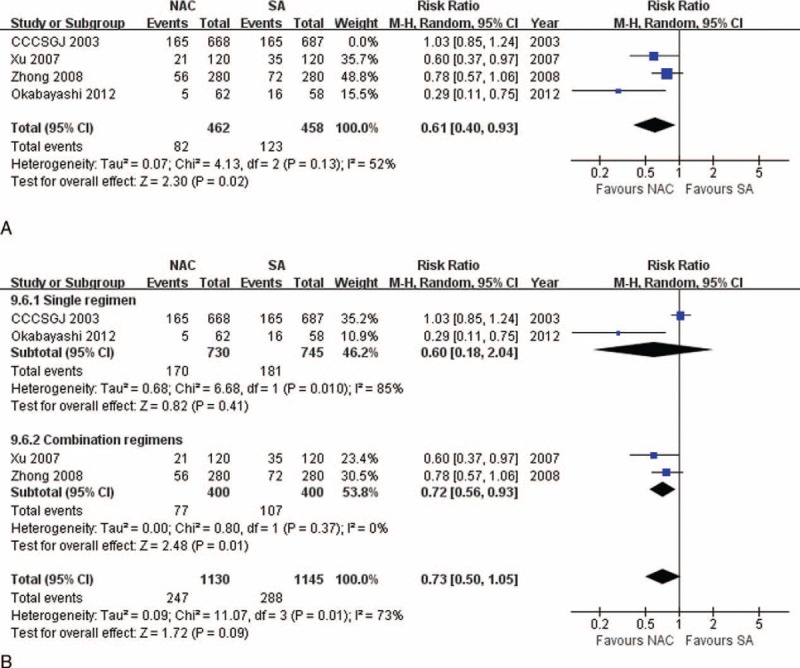
(A) Sensitivity test for total recurrence between NAC and SA measurements, showing that there exists significantly lower recurrence rate among patients receiving NAC than those undergoing SA when CCCSGJ's study was excluded. (B) Subgroup analysis for total recurrence according to whether single regimen or combination regimens are applied, showing that combination regimens significantly reduce overall recurrence rate, while single regimen makes no significant contribution. CCCSGJ = Colorectal Cancer Chemotherapy Study Group of Japan, NAC = neoadjuvant chemotherapy, SA = surgery alone.

There were significantly higher 3-year survival (86.26% vs 76.40%, RR: 1.13, 95% CI: 1.02–1.25, *P* = 0.02), and 3-year DFS (78.02% vs 64.04%, RR: 1.22, 95% CI: 1.06–1.39, *P* = 0.004) rates, and lower rates of overall recurrence (17.75% vs 26.86%, RR: 0.61, 95% CI: 0.40–0.93, *P* = 0.02, Figure 6A), and liver and lung metastases (15.25% vs 20.75%, RR: 0.73, 95% CI: 0.54–0.99, *P* = 0.04) for patients receiving NAC than those undergoing SA with CCCSGJ's study^20^ excluded. Sensitivity analyses of all the other indexes revealed similar results. Funnel plots (Figure 3A–G) and a strict and exhaustive literature retrieval conferred a substantial degree of confidence in our pooled findings.

### Subgroup Analysis

We divided subgroups according to whether single regimen or combination regimens were applied, and found that combination regimens used in NAC set significantly reduced overall recurrence rate compared with SA (19.25% vs 26.75%, RR: 0.72, 95% CI: 0.56–0.93, *P* = 0.01, Figure 6B).

## DISCUSSION AND CONCLUSIONS

Chemotherapy is a complementary treatment modality in the form of adjuvant chemotherapy, NAC and concomitant chemoradiotherapy.^25^ Potential advantages of preoperative therapy are making minimal access surgery practicable, better control of micrometastasis, and better tolerability than similar treatment after surgery, hence allowing increased dose intensity, and potentiality to downstage tumor and to increase the possibility of curative resection.^26^ There have been many trials assessing this novel method majorly among operable advanced CRC patients without distant metastasis and many reported satisfactory achievements.^26^ However, most of reports are limited to nonrandomized retrospective studies based on relatively small population.^27^ The optimal approach remains controversial.

The results of RCTs comparing NAC with SA for CRC differ in aspects of efficacy and safety. This study, ensuring high recall and precision rates of literature retrieval, summarizes data of the highest quality. RCTs published after 2005 constitute most of the studies included. The methodological quality of the 6 RCTs included in this meta-analysis was generally good.

Xu et al^21^ and Zhong et al^22^ reported that NAC in combination with surgical resection could reduce and delay the occurrence of liver metastasis, and improve survival rate in patients with stage III CRC; Okabayashi et al^23^ also reported that NAC might block recurrences and improve survival rates, mainly by preventing distant metastasis. The convincing level 1a evidence provided by us revealed that no significant differences existed in 3-year or 5-year OS or DFS, or total death, which may be due to the fact that NAC, though inhibiting malignant proliferation and promoting tumor necrosis, leads to attenuation of immunity and delay of timely curative treatment. Ishii et al's study^28^ demonstrated that preoperative chemotherapy had no impact on the prevention of local recurrence despite obvious tumor shrinkage in 46% of the participants. Our results further supported that NAC contributed to lower distant, especially liver, metastasis rate, but resulted in similar local recurrence rate. For stage I disease, timely surgery would be the ideal choice. In this analysis, patients with stage III disease had lower mortality, recurrence, and liver metastasis rates with NAC than SA, while in patients with stage II disease, the differences were not significant. This indicates that NAC could be beneficial to patients with diseases in advanced stages, which needs to be further clarified by larger sample size and longer follow-up period. Regimens and intervals between randomization/NAC application and operation might be factors potentially influencing efficacy and safety. It seems intuitively unlikely that such a short duration of chemotherapy applied in the included trials would significantly alter outcomes. Besides, thanks to the widespread high-quality surgery with satisfactory resections of regional lymph nodes outside the peri-colorectal area, there might be a better outcome than anticipated after curative SA, which could also conceal part of the effects. When CCCSGJ's results^20^ were excluded, we found that NAC resulted in significantly better outcomes, which might be because of the relatively inferior single regimen used, and the relatively long interval between randomization and surgery in the NAC arm. In CRC patients, combination therapies are related with a significant survival benefit compared to single agent therapy,^29^ and application of the most effective chemotherapeutic regimen is essential in the case of a NAC manipulation. However, single agent therapy was used in some of the RCTs available. Through a subgroup analysis, we revealed that combination therapy effectively impeded recurrence compared to single regimen, perhaps because of more effective micrometastasis eradication, reduced risk of incomplete excision, and tumor cell shedding during surgery after NAC.

NAC, which has been brought about with the hope to improve resection condition, is under heated discussion about its definite role in improving cure rate for CRC patients.^4^ According to our convincing study, better tumor conditions upon resection were screened with NAC applied, and NAC tended to prevent vascular invasion, which are the major differences between the 2 treatment modalities, while other advantages for resectability were not firmly supported. There were basically no significant differences in outcome measures of curative resection or postsurgical morbidities. It is notable that the current imaging technique available is not sufficiently accurate to assess the clinical staging of primary tumor or nodal stage, thus the exact down-staging effect could not be obtained, though other pre-treatment tumor parameters measurable were comparable between 2 groups. Lack of response to NAC may delay curative operation, and chemotherapy-related toxicity may lead to increased operational complications. The variability of the objective response rates may be influenced by issues of interval between administration and surgery, trial type and phase, regimen, and administration route. In a parallel audit,^30^ 93% of patients with radiologically classified T3 tumors with less than 5 mm invasion of the muscularis propria were found to have coexisting high-risk pathological features that justify chemotherapy. There also existed the risk of progression of similar chemoresistant tumors.^31^ Response rates higher than 50% are consistently achieved in metastatic CRC with chemotherapy regimens combining fluoropyrimidines with irinotecan or oxaliplatin,^32^ and even higher responses can be achieved in K-RAS wild-type tumors by adding EGFR-targeted monoclonal antibodies, panitumumab or cetuximab, to combination chemotherapy.^31^ Number of courses applied might be another great influential factor.

Though a number of phase III trials have been conducted in the last few decades, the best regimen of chemotherapy remains a point of argue and active research.^33^ Given the modern advancement of chemotherapy for CRC, a combination of chemotherapy and molecular targeting therapy may become mainstream.^34^ Recently, a number of new multicenter studies have been registered to assess the role of NAC in treating advanced CRC, and several new regimens are tested.^35^ It is desired that questions will be better addressed by them.

Therefore, NAC should not be recommended as a routine and regular treatment for CRC before gaining abundant evidence of its certain efficacy, and should be administered under the framework of clinical trials.^36^ Adequate operation (based on racial features, malignant progression, local standard, and surgeons’ experience) without delay may remain the appropriate and preferable management for resectable CRC, until further large multicenter RCTs supporting NAC emerges. Further investigations in terms of patient selection, and treatment regimen would be required to determine the significance of preoperative chemotherapy against CRC.

The internal validity of this study is high because the analysis was based on high-quality RCTs, with low risk of bias. This analysis is limited by the diverse timings, intervals between randomization and surgery, regimens, modalities of administration, durations of chemotherapy, and follow-up periods, and the fact that not all outcome measures of interest are reported by all selected trials.

In summary, NAC does not contribute to significant survival benefits for CRC, and compares favorably with SA in tumor-free resection rates, nodal status upon resection, and postsurgical complications. This might be due to the regimen issue. NAC significantly associates with favorable tumor stage upon resection, prevent distant (particularly liver) metastasis, and may result in lower incidences of vascular invasion and overall recurrence. This level 1a evidence does not support NAC to obviously outweigh SA in terms of efficacy and safety for CRC.
